# Comparing High- and Low-Model for End-Stage Liver Disease Living-Donor Liver Transplantation to Determine Clinical Efficacy: A Systematic Review and Meta-Analysis (CHALICE Study)

**DOI:** 10.3390/jcm12185795

**Published:** 2023-09-06

**Authors:** Kumar Jayant, Thomas G. Cotter, Isabella Reccia, Francesco Virdis, Mauro Podda, Nikolaos Machairas, Ramesh P. Arasaradnam, Diego di Sabato, John C. LaMattina, Rolf N. Barth, Piotr Witkowski, John J. Fung

**Affiliations:** 1 Department of Surgery and Cancer, Hammersmith Hospital, Imperial College London, London W12 0TS, UK; 2 Department of General Surgery, Memorial Healthcare System, Pembroke Pines, FL 33028, USA; 3 Division of Digestive and Liver Diseases, UT Southwestern Medical Center, Dallas, TX 75390, USA; 4 General Surgery and Oncologic Unit, Policlinico ponte San Pietro, 24036 Bergamo, Italy; isabella.reccia@gmail.com; 5 Dipartimento DEA-EAS Ospedale Niguarda Ca’ Granda Milano, 20162 Milano, Italy; 6 Department of Surgery, Calgiari University Hospital, 09121 Calgiari, Italy; 7 2nd Department of Propaedwutic Surgery, National and Kapodistrian University of Athens, 11527 Athens, Greece; nmachair@gmail.com; 8 Warwick Medical School, University of Warwick, Coventry CV4 7H, UK; 9 The Transplantation Institute, Department of Surgery, University of Chicago, Chicago, IL 60637, USA

**Keywords:** liver transplant, living donor, high MELD, living donor liver transplant (LDLT)

## Abstract

Introduction: Various studies have demonstrated that low-Model for End-Stage Liver Disease (MELD) living-donor liver transplant (LDLT) recipients have better outcomes with improved patient survival than deceased-donor liver transplantation (DDLT) recipients. LDLT recipients gain the most from being transplanted at MELD <25–30; however, some existing data have outlined that LDLT may provide equivalent outcomes in high-MELD and low-MELD patients, although the term “high” MELD is arbitrarily defined in the literature and various cut-off scores are outlined between 20 and 30, although most commonly, the dividing threshold is 25. The aim of this meta-analysis was to compare LDLT in high-MELD with that in low-MELD recipients to determine patient survival and graft survival, as well as perioperative and postoperative complications. Methods: Following PROSPERO registration CRD-42021261501, a systematic database search was conducted for the published literature between 1990 and 2021 and yielded a total of 10 studies with 2183 LT recipients; 490 were HM-LDLT recipients and 1693 were LM-LDLT recipients. Results: Both groups had comparable mortality at 1, 3 and 5 years post-transplant (5-year HR 1.19; 95% CI 0.79–1.79; *p*-value 0.40) and graft survival (HR 1.08; 95% CI 0.72, 1.63; *p*-value 0.71). No differences were observed in the rates of major morbidity, hepatic artery thrombosis, biliary complications, intra-abdominal bleeding, wound infection and rejection; however, the HM-LDLT group had higher risk for pulmonary infection, abdominal fluid collection and prolonged ICU stay. Conclusions: The high-MELD LDLT group had similar patient and graft survival and morbidities to the low-MELD LDLT group, despite being at higher risk for pulmonary infection, abdominal fluid collection and prolonged ICU stay. The data, primarily sourced from high-volume Asian centers, underscore the feasibility of living donations for liver allografts in high-MELD patients. Given the rising demand for liver allografts, it is sensible to incorporate these insights into U.S. transplant practices.

## 1. Introduction

Given the context of increased disease prevalence, expanding indications for liver transplantation (LT) and the shortage of deceased-donor liver allografts, living-donor liver transplantation (LDLT) has emerged as an option to bridge the disparity between demand and requirement and scale down waitlist mortality. Adult-to-adult LDLT comprises ~7% of all 2019 adult LTs in the U.S., and overall, less than 30% of all LTs in the Americas and Europe [1,2]. Although the 2019 LDLT figure represents the highest annual number of LDLTs performed in the U.S since 2001, the expansion of LDLT practice has previously been impacted by the rapid growth of deceased-donor LT (DDLT), and donors’ ethical concerns have been exacerbated by an unfortunate donor death in 2001, which prompted the implementation of more stringent LDLT regulations [3,4]. Moreover, the New York state committee on quality improvement recommended that LDLT should not be offered to patients with MELD (Model for End-Stage Liver Disease) scores above 25 (High MELD) [3]. However, these guidelines are amenable to modification in light of an availability of larger data and better evidence.

In spite of patients with high MELD scores receiving priority treatment in the current organ allocation system in the U.S. and Europe, a substantial proportion of patients may still not receive a life-saving organ in an expeditiously sufficient fashion, particularly those with a MELD score below 30. Moreover, the 90-day mortality risk of patients with acute-on-chronic liver failure can be underestimated in patients, and thus, these patients may not receive sufficient organ offers [5]. In contrast to DDLT, LDLT provides a healthy part of a donor liver with minimal ischemic time in an expeditious fashion during an elective surgery [6,7,8].

Even in the modern era, the experiences and outcomes of LDLT patients continue to be differentiated between lower-volume, Western hemisphere countries and high-volume programs from the Middle East and Asia that rely on LDLT to overcome cultural and religious barriers to DDLT. With increasing experience, LDLT has been shown to result in equivalent outcomes in high-MELD patients, and in some cases, superior recipient survival and long-term outcomes compared to deceased-donor LT. The latest LDLT patient outcomes are indeed excellent despite the technically demanding aspects of the surgery, requiring appropriately high surgical volumes on a regular basis to assure appropriate competency [2]. Despite these advantages of utilizing LDLT, there is a paucity of literature on such outcomes in the high-MELD LT candidate population. Hence, a strong argument can be made that LDLT should be considered as an alternative to DDLT irrespective of MELD score.

Although the traditional literature suggests that LDLT recipients gain the most from being transplanted at MELD < 25, there are some contemporary data that also highlight that LDLT may provide equivalent outcomes in high-MELD recipients. Lee et al. (2012) compared the outcomes of LDLT and DDLT patients with MELD > 30. Here, the authors reported an improved OS following LDLT, even in instances with hepatorenal syndrome [9]. Similarly, high-volume centers in Taiwan and India have reported comparable 5-year overall survival for LDLT patients with MELD > 30 to candidates transplanted at MELD < 30 [10].

The objective of this study was to perform a cumulative, world-wide analysis of outcomes comparing LDLT in high-MELD recipients to those of low-MELD recipients to deduce overall patient survival, graft survival and perioperative outcomes.

## 2. Methods

### 2.1. Literature Search Methodology

We performed a systematic literature search of articles indexed in PubMed, EMBASE, Cochrane, Crossref, Scopus and clinical trial registries. Our search strategy was based on recommendations from the *Cochrane Handbook for Systematic Reviews of Intervention* and reported according to the guidelines of *Meta-analysis of Observational Studies in Epidemiology* [11,12]. The MeSH terms and free terms ‘liver’ or ‘hepatic’ and ‘transplants’ or ‘liver transplantation’ or ‘liver transplant’ and ‘living donor’ or ‘live donor’ and ‘high MELD’ and ‘low MELD’ were combined, and additional free text searches were carried out as required. The initial database search was performed on 25 October 2021, without any limitations, including date, study type, language, or any other limiting attributes. Additional studies were identified via a manual search of preprints, case reports, abstracts, bibliographies and a citation list of relevant articles using the free search terms. This study was registered in PROSPERO, which is an international database of prospectively registered systematic reviews (CRD-42021261501).

### 2.2. Inclusion and Exclusion Criteria for Study Selection

All available studies in the literature pertaining to the topic of interest were included. However, the term “high” MELD is arbitrarily defined in the literature and various cut-off scores are outlined between 20 and 30, although the most commonly dividing threshold is 25. Hence, we did include all relevant studies comparing post-transplant ≥1-year overall survival outcomes following LDLT in high- and low-MELD recipients aged ≥ 18. Studies involving re-transplants and multiorgan-transplants were not included. Further, all other publications, such as editorials, letters, reviews, case reports and articles with duplicate data, were excluded.

### 2.3. Data Extraction and Statistical Analysis

The selected studies were reviewed by two separate physician reviewers, KJ and TC, who employed a two-stage method to independently screen the identified articles using a shared online form. In cases of discrepancy and inconsistencies, items were resolved through discussion and mediation by the chief author, JF, serving as the arbitrator. We extracted the following data from included studies: study characteristics (year of publication, first author, country), baseline demographics and liver disease diagnosis. The primary studied outcomes were 1-, 3- and 5-year patient survival. The analyzed secondary outcomes were 1-, 3- and 5-year graft survival and operative time and postoperative variables (biliary complications, hepatic artery thrombosis (HAT), infection, rejection and length of stay). The postoperative complications were compiled according to the Clavien–Dindo classification [13].

For the meta-analysis, percentages and total numbers were used to report categorical variables, and mean with standard deviation (SD) for continuous variables. When the included studies reported the median and interquartile range, the mean and SD were estimated according to established methods. Continuous variables were analyzed using mean difference (MD), whereas categorical variables were analyzed using odds ratio (OR), both with 95% confidence intervals (CI). In cases where mean values and standard deviations were not reported, the values were determined according to mathematical equations outlined by Hozo et al. [14].

The hazard ratio (HR) for time-to-event outcomes was estimated indirectly from other summary statistics or from data in published Kaplan–Meier curves [15]. The expected number of cases (O-E) and the variance for the single studies were utilized in the estimation of individual and overall HR using a fixed-effects model to ascertain a pooled HR in order to complete the survival analyses [16]. All the data analyses were performed through the random-effects model in STATA/SE 16 (Stata, College Station, TX, USA) and RevMan 5.3 as required. The risk of bias for each included study was assessed according to the quality assessment tool published by the National Institutes of Health (i.e., Quality Assessment Tool for Case Series Studies), which is the preferred tool for assessments of risk of bias in systematic reviews registered in the PROSPERO protocols [17,18]. Heterogeneity among the included studies was investigated using I^2^-statistics and classified in the following fashion: I^2^ of ≤25% was interpreted as low heterogeneity; I^2^ of 25–75% indicated moderate heterogeneity, and ≥75% was interpreted as high heterogeneity. The random-effects model was adopted to balance intrinsic heterogeneity and effect size [19,20].

## 3. Results

### 3.1. Review of the Literature

An initial literature review yielded 656 abstracts, and full-text review was performed for 117 articles.

**Figure 1:**
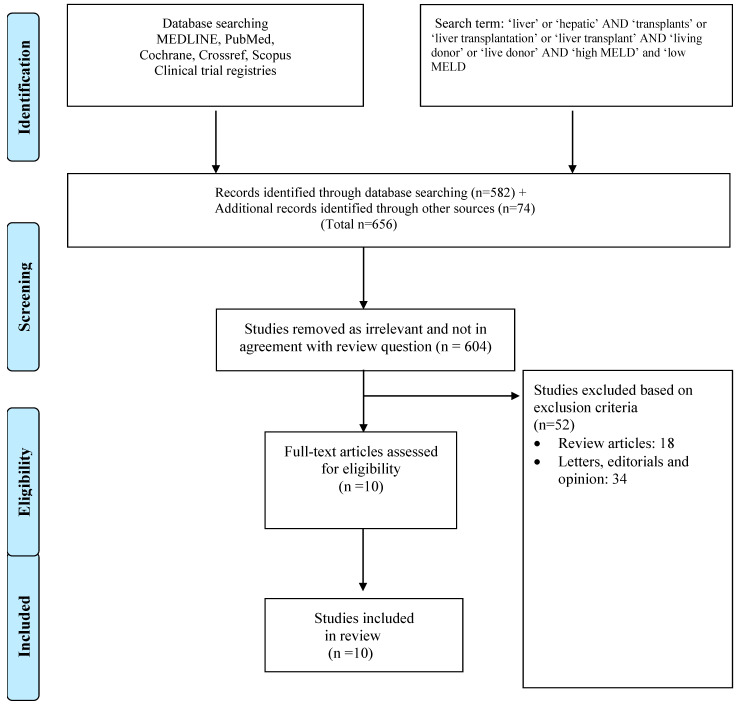
Search strategy and study selection used in this systematic review as per PRISMA protocol.

**Figure 2:**
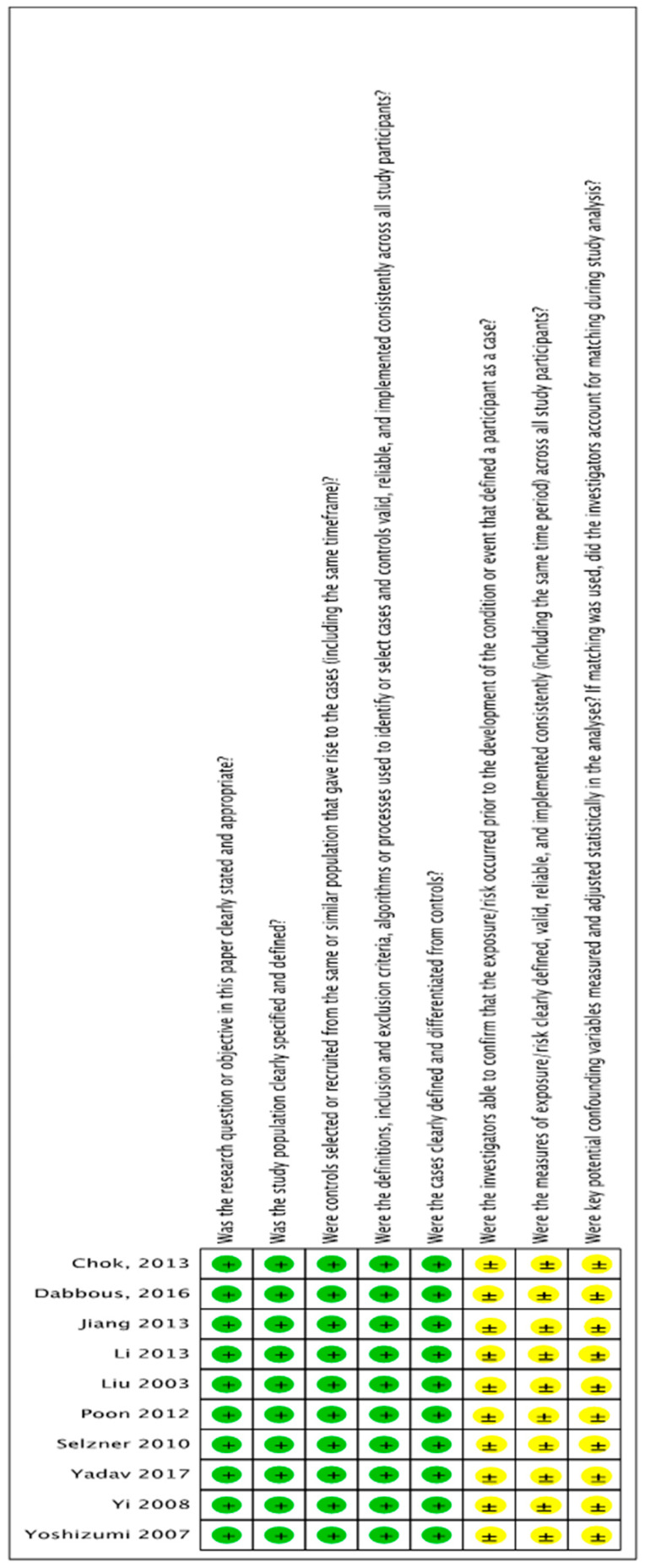
Quality assessment of included studies. Green present; yellow equivocal [21,22,23,24,25,26,27,28,29,30].

Figure 1 outlines the PRISMA flowchart and search results at each stage of evaluation as per the study criteria, and the studies selected for data extraction. A total of 10 articles from various nations, including Canada, Japan, China, Taiwan, Korea and India, were identified for meta-analyses [21,22,23,24,25,26,27,28,29,30]. All studies were retrospective observational cohort studies, and the most commonly dividing threshold of MELD was 25, except in two studies, where cut-off values were 20 and 30, respectively (Table 1) [25,29]. The Quality Assessment Tool for Case Series Studies showed that the nature of the included studies was fair or good (Figure 2).

### 3.2. Patient Population Characteristics

A total of 10 studies with 2183 LT recipients met the predefined selection criteria: 490 were high-MELD living-donor liver transplant (HM-LDLT) recipients and 1693 were low-MELD living-donor liver transplant (LM-LDLT) recipients. The HM-LDLT cohort had a significantly younger population with a mean age of 42.26 vs. 48.36 in the LM-LDLT recipients (MD −3.43; 95% CI −6.02, −0.85; *p*-value 0.009). Both groups were comparable in terms of sex distribution (*p*-value 0.26). The most common transplant indication was viral hepatitis (49.2%), followed by HCC (21.5%), alcoholic liver disease (14.9%) and cholestatic liver disorders (13.2%) (Table 1 and Table 2).

### 3.3. Preoperative Characteristics

The following preoperative variables were assessed: ascites, hepatic encephalopathy (HE) and serum bilirubin. As expected, there were statistically significantly higher rates of ascites and HE (OR 4.35; 95% CI 2.41, 7.84; *p*-value 0.000) among the HM-LDLT recipients compared to the LM-LDLT cohort. Serum bilirubin level was also significantly higher in the HM-LDLT recipients when compared to the LM-LDLT recipients (MD 17.39; 95% CI 2.20, 32.59; *p*-value 0.02) (Table 2 and Table 3). Further, we did analyze the graft-to-recipient weight ratio (GRWR) and graft weight-to-recipient estimated standard liver weight (GW/ RESLW) and observed no statistically significant difference (MD −0.02; 95% CI −0.05, 0.01; *p*-value 0.16 and MD −1.41; 95% CI −6.79, 3.98; *p*-value 0.61, respectively).

### 3.4. Survival Outcomes

**Figure 3:**
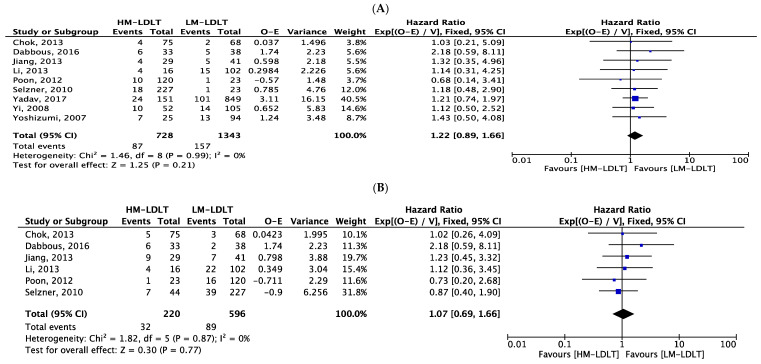

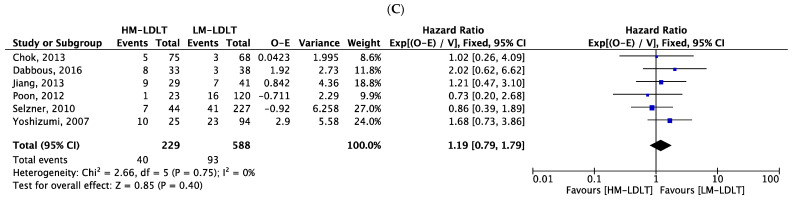
Forest plot depicting hazard ratios for overall patient survival at 1 year (**A**), 3 years (**B**) and 5 years (**C**) post-transplant, demonstrating equivalent outcomes in HM-LDLT and LM-LDLT recipients [22,23,24,25,26,27,28,29,30].

**Figure 4:**
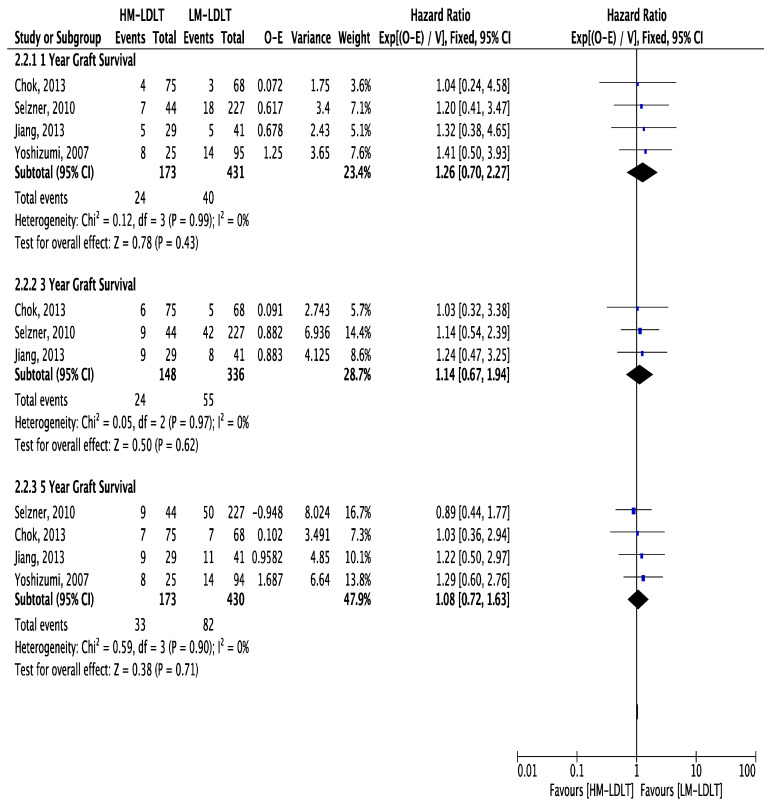
Forest plot depicting hazard ratios for overall graft survival at 1 year (**A**), 3 years (**B**) and 5 years (**C**) post-transplant, demonstrating equivalent outcomes in HM-LDLT and LM-LDLT recipients [22,24,26,27].

The primary outcome of interest was to determine and compare overall patient survival between the studied cohorts (Table 4). Based upon the available data, patient survival following transplantation between the HM-LDLT and LM-LDLT recipients was comparable at 1 year (HR 1.22; 95% CI 0.89, 1.66; *p*-value 0.21), 3 years (HR 1.07; 95% CI 0.69, 1.66; *p*-value 0.77) and 5 years (HR 1.19; 95% CI 0.79, 1.79; *p*-value 0.40) (Figure 3). Graft survival was also comparable at 1 year (HR 1.26; 95% CI 0.70, 2.27; *p*-value 0.43), at 3 years (HR 1.14; 95% CI 0.67, 1.94; *p*-value 0.62) and at 5 years (HR 1.08; 95% CI 0.72, 1.63; *p*-value 0.71) (Figure 4).

### 3.5. Perioperative Outcomes

Here, we analyzed perioperative outcomes as secondary outcomes among the study groups, such as operative time, morbidity, postoperative complications and intensive care unit and hospital stay (Table 4). Based on the available data from seven studies, there was no difference in operating time between the studied cohorts (MD 14.69; 95% CI −31.57, 2.19; *p* = 0.09; I^2^ 27.14%). Major morbidity (Clavien–Dindo grade ≥ III) was reported in five studies and no statistically significant difference was found between both groups (OR 1.53; 95% CI 0.92, 2.54; *p* = 0.10; I^2^ 41%). There was also no increased risk of HAT in either group (OR 1.18; 95% CI 0.52, 2.67; *p* = 0.70; I^2^ 0%). Biliary complications were outlined in seven studies, and we did not observe any statistically significant differences between the groups (OR 1.06; 95% CI 0.72, 1.57; *p* = 0.75; I^2^ 14%).

The risk of pulmonary infection was significantly higher in the HM-LDLT vs. the LM-LDLT group (OR = 2.07; 95% CI 1.05, 4.08; *p* = 0.04; I^2^ 40%); however, there was no difference in wound infection rates (OR = 1.46; 95% CI, 0.51, 4.24; *p* = 0.48; I^2^ 0%).

Six out of ten studies reported intra-abdominal bleeding and there was no significant difference between the studied groups (OR = 1.87; 95% CI, 0.98, 3.54; *p* = 0.06; I^2^ 0%). However, our meta-analysis demonstrated a markedly higher rate of abdominal fluid collection in HM-LDLT recipients compared to LM-LDLT recipients (OR = 2.26; 95% CI, 1.21, 4.24; *p* = 0.01; I^2^ 0%). Additionally, our analysis showed no increased risk of rejection in either group HM-LDLT or LM-LDLT (OR = 1.18; 95% CI, 0.80, 1.75; *p* = 0.41; I^2^ 0%).

**Figure 5:**
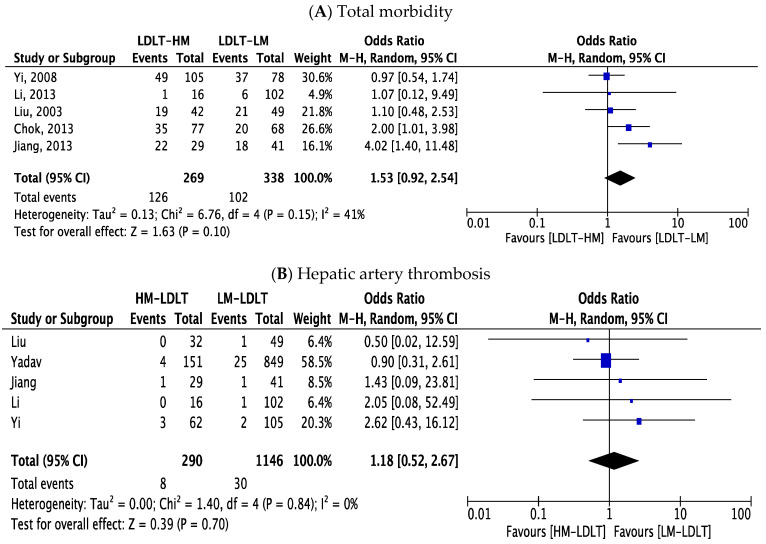

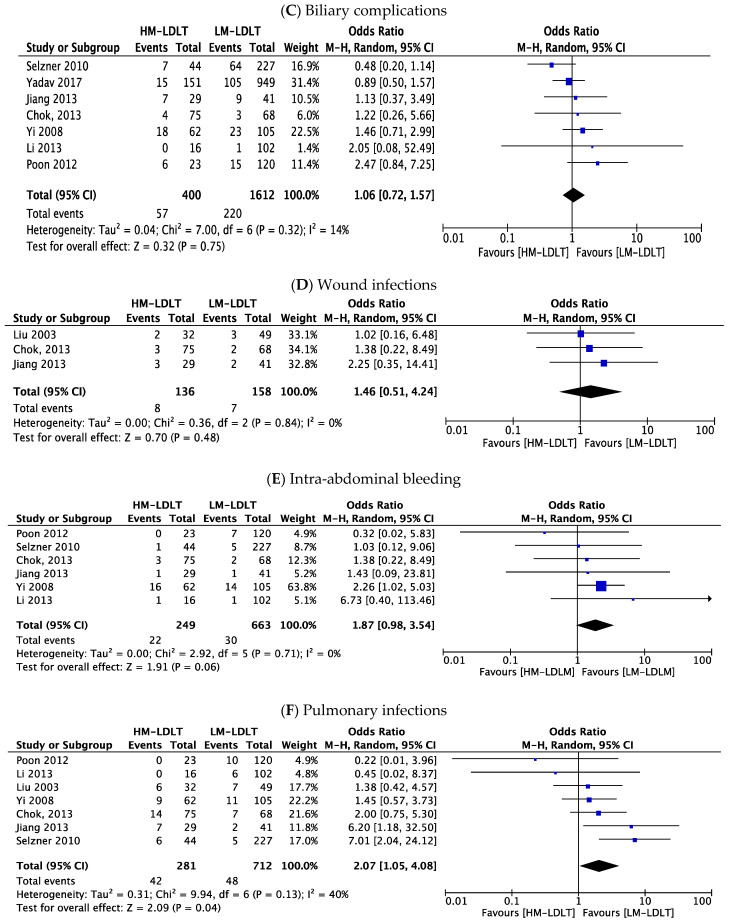

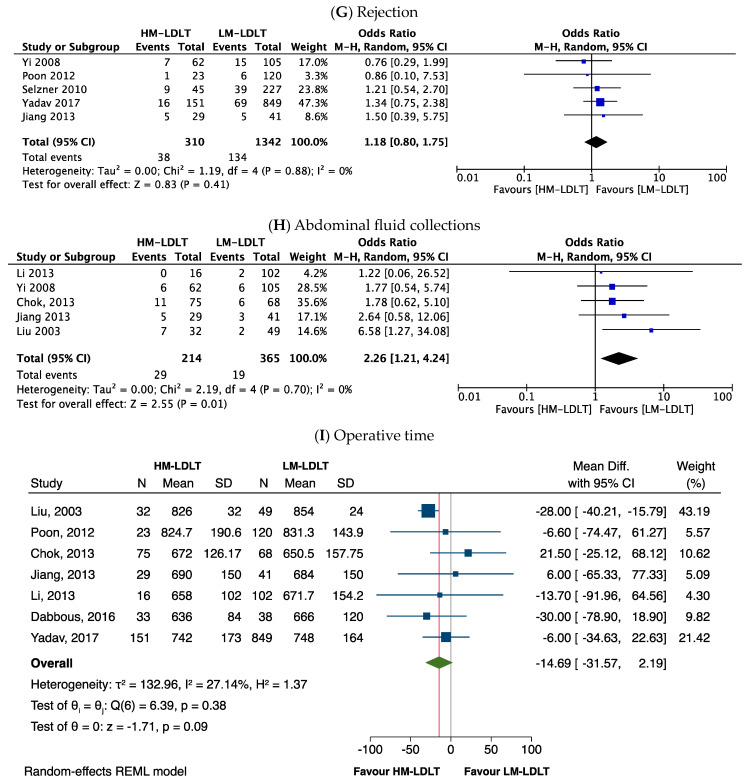

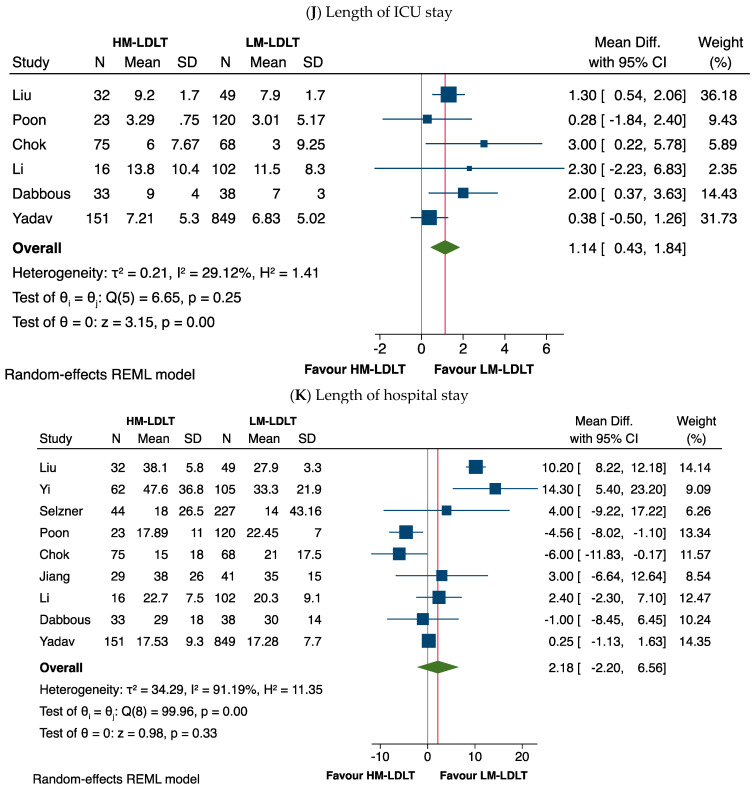
Forest plot of operative and postoperative variables. (**A**) Total morbidity, (**B**) hepatic artery thrombosis, (**C**) biliary complications, (**D**) wound infection, (**E**) intra-abdominal bleeding, (**F**) pulmonary infection, (**G**) abdominal fluid collection, (**H**) rejection, (**I**) operative time, (**J**) length of ICU stay, (**K**) length of hospital stay. HM-LDLT and LM-LDLT recipients had equivalent rates of total morbidity (**A**), hepatic artery thrombosis (**B**), biliary complications (**C**), wound infection (**D**) intra-abdominal bleeding (**E**), rejection (**G**) and length of hospital stay; however, HM-LDLT recipients had higher likelihood of pulmonary infection (**F**), abdominal fluid collection (**G**) and prolonged ICU stay [21,23,24,25,26,27,28,29,30].

Length of ICU stay was outlined in six studies and found to be significantly shorter in the LM-LDLT group than the HM-LDLT group (MD 1.14; 95% CI 0.43, 1.84; *p* = 0.00; I^2^ 29.12%); however, overall hospital stay was comparable (MD 2.18; 95% CI −2.20, 6.56; *p* = 0.33; I^2^ 91.19%), though the heterogeneity was significant (Figure 5).

## 4. Discussion

Owing to the advancement of surgical techniques and technology over the last two decades, LDLT might be offered to the most patients on current waitlist, which could not only increase the number of transplantations but also help in reducing waiting list mortality. Hence, LDLT may be a suitable option for decompensated liver disease patients who are in need of expeditious transplantation [31,32]. Our meta-analysis = assessed a world-wide population of 490 HM-LDLT and 1693 LM-LDLT patients with a broad range of liver disorders. The primary outcome of our study and analysis showed that the overall patient survival at 1-, 3- and 5-years was equivalent in the HM-LDLT and LM-LDLT groups. Similarly, HM-LDLT offered comparable graft survival when compared to LM-LDLT at all the studied time periods. Owing to being in a state of heightened disease severity for liver disease, the observed incidence of preoperative ascites, encephalopathy and serum bilirubin level was significantly higher in the HM-LDLT group. Further, despite a higher incidence of postoperative pulmonary infection, higher intra-abdominal fluid collection and prolonged ICU stays, HM-LDLT recipients had comparable operative time and a similar incidence of HAT, biliary complications, would infection, intra-abdominal bleeding, rejection, overall morbidity and overall hospital stays when compared to LM-LDLT.

To our knowledge, our study is the first meta-analysis comparing the clinical and survival outcomes of HM-LDLT recipients in contrast to LM-LDLT recipients, and outlines a broader relative review of the end results. Collectively, this analysis demonstrated comparable outcomes and suggests that the advantages of LDLT in low-MELD patients can be safely extended to high-MELD patients with similar morbidity and survival outcomes.

LDLT is a complex surgery, requiring a high level of surgical skills and advanced perioperative medical care. Therefore, a considerable risk exists for perioperative complications such as vascular complications, biliary stricture or leak, early graft dysfunction or early allograft loss necessitating re-transplant. Nevertheless, several studies emanating from the experienced centers of the world have outlined equivalent or improved graft survival outcomes after LDLT compared to DDLT irrespective of the consideration of MELD score [33,34,35,36,37,38,39]. Historically, the reported incidence of vascular complications following LDLT was up to 9%; however, with increasing experience in LDLT, the reported incidence in the contemporary literature is comparable to the incidence in DDLT in both high- and low-MELD recipients [8]. The incidence of HAT in our data analysis was as low as 2.7% and 2.6% following LDLT in high- and low-MELD patients. Further, comparative analysis reported no statistically significant difference in HAT incidence among both groups.

Biliary complications have been constantly implicated as important cause of increased morbidity and mortality following LT [40,41,42]. Recently, a retrospective study was performed to demonstrate the incidence of biliary complications in LT recipients. The reported incidence of biliary complication in LDLT and DDLT recipients was 17.3% and 18.7%, respectively, and a univariate analysis outlined various risk factors for biliary complications, including multiple bile duct anastomosis and recurrent cholangitis before transplantation [43]. Moreover, a review of the contemporary literature on heightened biliary complication has outlined several other attributes such as prolonged cold ischemia time, MELD > 35, biliary leak and HAT [44]. The observed incidence of biliary complication in the current study was 14.2% in HM-LDLT patients and 13.6% in LM-LDLT patients, and they were statistically comparable.

Pulmonary complications are another major reason for post-transplant morbidity and mortality and range from 8.8 to 43.36% [45,46]. The most commonly listed risk factors include age, high MELD score, increased severity of liver disease, a higher incidence of restrictive lung disease in high-MELD patients, uncontrolled ascites, an underlying lung disorder, associated renal failure necessitating hemodialysis and the requirement of mechanical ventilation prior to transplant [47,48,49]. Our meta-analysis confirmed high MELD as a major risk factor for heightened postoperative pulmonary infections with significantly higher incidence in HM-LDLT (14.9%) compared to LM-LDLT patients (6.7%). Moreover, the meta-analysis demonstrated prolonged ICU stays in HM-LDLT recipients when compared to LM-LDLT recipients. As expected, a higher incidence of abdominal fluid collection following LT was observed in high-MELD patients with uncontrolled ascites. Abdominal fluid collections such as hematomas, seromas, bilomas, localized ascites and abscesses can impact graft survival, but we did not observe such predicaments in this index study.

Multitudes of studies have demonstrated a higher rate of bacterial infection among DDLT recipients in contrast to LDLT recipients, and deceased-donor allograft is recognized as an independent risk factor for higher post-operative infection rate. Postoperative infections are also considered one of the major causes of postoperative morbidity and mortality [44,50,51,52]. Interestingly, our meta-analysis reported comparable outcomes among HM-LDLT and LM-LDLT recipients in terms of wound infections, postoperative morbidity and overall hospital stay.

The clear benefits of living donation are the avoidance of prolonged cold ischemia time and related significant ischemic reperfusion injury of the deceased-donor liver allograft. Further, researchers have postulated that the donor body is also under significant duress during donation after brain death (DBD) owing to the “autonomic storm” and profound inflammatory response, thereby disrupting liver immune homeostasis [53]. Additionally, a liver allograft following donation after circulatory death is exposed to an extended duration of warm ischemia along with an intense inflammatory response, thus limiting the intended benefits of liver transplantation. On the contrary, liver allografts following living donation are less exposed to such stresses, despite the associated caveat that a lower liver mass is provided in living grafts compared to full deceased grafts. Several studies have reported lower rates of rejection in LDLT recipients compared to DDLT recipients [54,55,56]. Our meta-analysis shows comparable rejection risk among both groups, and hence, does not outline any untoward effect of a high MELD score in terms of allograft rejection.

The practice of high-MELD LDLT has been more prevalent in large-volume centers in Asia, whereby LDLT is the most pragmatic approach to LT given the cultural and religious barriers towards DDLT. Further, the increasing worldwide cumulative experience in LDLT has ushered in changes in the management protocol of end-stage liver disease with expedited LT to high-MELD candidates. Our review of the contemporary literature showed equivalent outcomes following LDLT in high-MELD patients, and in some cases, superior recipient survival and long-term outcomes compared to deceased-donor LT [57,58,59,60]. Hence, offering LDLT to high-MELD candidates appears to be a feasible approach to expanding the donor pool, if significant LDLT expertise is achieved.

Overall, our analysis revealed excellent primary outcomes (patient and graft survival rates) with no difference among high-MELD and low-MELD LDLT recipients, and based upon our review of the existing literature, they were comparable to DDLT patients’ outcomes.

In a recent meta-analysis, Barbetta et al. (2020) compared the clinical outcomes of LDLT patients with those of deceased-donor LT (DDLT) patients. The authors included 19 international studies, with 4571 LDLT and 66,826 DDLT recipients, and concluded improved patient survival, lower waiting times and similar graft survival in the LDLT cohort [8]. Whilst LDLT recipients gain the most from being transplanted at MELD <25, studies have demonstrated fairly reasonable outcomes following LDLT in high-MELD candidates. Lee et al. (2012) compared the outcomes of LDLT and DDLT patients with MELD >30. The authors reported improved overall survival (OS) following LDLT, even in instances of hepatorenal syndrome [61]. Similarly, high-volume centers in Taiwan and India have reported comparable 5-year OS for LDLT in high-MELD candidates in contrast to low-MELD score recipients [25,30]. In addition, a living-donor liver allograft is not exposed to brain death, which not only alters the quality of the donor organ but may also negatively affect both graft and patient survival [62,63].

There were certain limitations of this meta-analysis that should be understood in the context of the data available in the included studies. Firstly, the included studies were retrospective in nature, and despite having balanced baseline characteristics, there might be some confounders that may influence the outcomes. Additionally, the reported clinical trajectories, management approaches and outcomes were likely to differ owing to the availability of resources and demographics in that geographical area; however, considering the paucity of high-quality evidence in the index field, meta-analyses of non-randomized studies could be of enormous value. It needs to be highlighted that our meta-analysis included publications from the high-volume LDLT center; however, the results are replicable in other centers with growing experience. Despite some of these caveats, the strengths of this meta-analysis were the extensive and exhaustive nature of the search, the independent process of study selection and data abstraction, and the random-effects model being applied during pooled data analysis to limit the shadow of heterogeneity, though for most of the instances, the observed heterogeneity was mild to moderate.

## 5. Conclusions

This meta-analysis was based on data from the most experienced LDLT centers and demonstrated an equivalent survival benefit of LDLT among high-MELD and low-MELD patients. The evidence gathered has clearly demonstrated that the safety and benefits of LDLT in low-MELD patients can be extended to high-MELD patients with similar morbidity and survival outcomes; thus, living donation should be considered as an optimal means of expanding the donor pool, reducing waitlist mortality and potentially conferring better long-term outcomes compared with DDLT. Deriving predominantly from high-volume living-donor liver transplant centers, the data elucidate the intricacies and viability of living donations for liver allografts in patients with elevated MELD scores. Given this backdrop, there emerges an imperative to assimilate these revelations into the prevailing paradigms of transplant methodologies within the U.S. patient populace.

## Figures and Tables

**Table 1:** Characteristics of included studies in HM and LM living-donor liver transplant recipients.

Study	Country	High-MELD–Low-MELD Cut-Off	Study Arms (n)	Age, Years (Mean ± SD)	Sex, Male, n (%)	Diagnosis			
						HCC	HCV/HBV	ALD	PSC/PBC/AIH/CC
Liu, 2003 [21]	China	25	HM-LDLT (32)	40.6 ± 1.8	26 (81.3%)			_	
			LM-LDLT (49)	48.5 ± 1.1	42 (85.7%)		44		5
Yoshizumi, 2007 [22]	Japan	25	HM-LDLT (25)	43.2 ± 12.8	11 (44.0%)				
			LM-LDLT (94)	52.5 ± 11.3	53 (56.4%)				
Yi, 2008 [23]	Korea	25	HM-LDLT (62)	47.7 ± 8.9	46 (74.2%)				
			LM-LDLT (105)	49.5 ± 7.7	75 (71.4%)	64	48		
Selzner, 2010 [24]	Canada	25	HM-LDLT (44)	49 ± 10	26 (59.1%)				
			LM-LDLT (227)	51 ± 10	129 (56.8%)	61	93		
Poon, 2012 [25]	Taiwan	30	HM-LDLT (23)	54.1 ± 7.28	15 (65.2%)				
			LM-LDLT (120)	49.7 ± 8.95	94 (78.3%)		96	14	10
Chok, 2013 [26]	China	25	HM-LDLT (75)	45.5 ± 8.0	27 (36.0%)				
			LM-LDLT (68)	45.8 ± 12.3	21 (30.9)		50		9
Jiang, 2013 [27]	China	25	HM-LDLT (29)	38.5 ± 7.7	25 (86.2%)				
			LM-LDLT (41)	41.5 ± 8.4	37 (90.2%)		41		
Li, 2013 [28]	China	25	HM-LDLT (16)	37.6 ± 7.7	8 (50%)				
			LM-LDLT (102)	44 ± 8.2	94 (92.2%)	68	90		
Dabbous, 2016 [29]	Egypt	20	HM-LDLT (33)	46.2 ± 7.9	32 (97%)	11			
			LM-LDLT (38)	47.8 ± 7.8	34 (89.5%)	11			
Yadav, 2017 [30]	India	25	HM-LDLT (151)	43.6 ± 9.74	125 (82.8%)				
			LM-LDLT (849)	50.3 ± 9.81	698 (82.2%)	226	376	260	213

**Table 2:** Preoperative attributes in the study groups.

Attributes	Number of Studies	MD	CI (95%)	*p*-Value	I^2^ (%)	Remarks
**Age (recipient)**	10	−3.43	−6.02, −0.85	0.009 **	91%	Significantly younger population in HM group
**Preoperative bilirubin (mg/dL)**	3	17.39	2.20, 32.59	0.02 **	99%	Significantly higher bilirubin in HM group
**GRWR**	4	−0.02	−0.05, 0.01	0.16	0%	No significant difference
**GW/R ESLW**	3	−1.41	−6.79, 3.98	0.61	92%	No significant difference

GW/R ESLW: graft weight/recipient estimated standard liver weight; GRWR: graft/recipient weight ratio; MD: mean difference; CI: confidence interval. ** significant.

**Table 3:** Preoperative attributes in the study groups.

Attributes	Number of Studies	OR	CI (95%)	*p*-Value	I^2^ (%)	Remarks
**Male**	10	0.78	0.51, 1.19	0.26	56%	No significant difference
**Ascites**	3	2.72	1.57, 4.71	0.000 **	0%	Significantly higher incidence in HM group
**Encephalopathy**	3	4.35	2.41, 7.84	0.000 **	0%	Significantly higher incidence in HM group

OR: odds ratio; CI: confidence interval. ** significant.

**Table 4:** Pooled estimates of outcomes and adverse events in HM group vs. LM group using random-effects meta-analysis.

A. Outcomes	Odds Ratio	95% CI	*p*-Value	I^2^ (%)
Major morbidity	1.53	0.92–2.54	0.10	41%
Hepatic artery thrombosis	1.18	0.52–2.67	0.70	0%
Biliary complications	1.06	0.72–1.57	0.75	14%
Wound infections	1.46	0.51–4.24	0.48	0%
Intra-abdominal bleeding	1.87	0.98–3.54	0.06	0%
Pulmonary infections	2.07	1.05–4.08	0.04 *	40%
Abdominal fluid collection	2.26	1.21–4.24	0.01 *	0%
**B. Outcomes**	**Mean difference**	**95% CI**	***p*-Value**	**I^2^ (%)**
Length of ICU stay	1.14	0.43–1.84	0.00 *	29%

* significant.

## Data Availability

All data that support the conclusions of this manuscript are included within the article.
